# Prokayrotic Ubiquitin-Like Protein (Pup) Proteome of *Mycobacterium tuberculosis*

**DOI:** 10.1371/journal.pone.0008589

**Published:** 2010-01-06

**Authors:** Richard A. Festa, Fiona McAllister, Michael J. Pearce, Julian Mintseris, Kristin E. Burns, Steven P. Gygi, K. Heran Darwin

**Affiliations:** 1 Department of Microbiology, New York University School of Medicine, New York, New York, United States of America; 2 Department of Cell Biology, Harvard Medical School, Boston, Massachusetts, United States of America; University of Delhi, India

## Abstract

Prokaryotic ubiquitin-like protein (Pup) in *Mycobacterium tuberculosis* (*Mtb*) is the first known post-translational small protein modifier in prokaryotes, and targets several proteins for degradation by a bacterial proteasome in a manner akin to ubiquitin (Ub) mediated proteolysis in eukaryotes. To determine the extent of pupylation in *Mtb*, we used tandem affinity purification to identify its “pupylome”. Mass spectrometry identified 55 out of 604 purified proteins with confirmed pupylation sites. Forty-four proteins, including those with and without identified pupylation sites, were tested as substrates of proteolysis in *Mtb*. Under steady state conditions, the majority of the test proteins did not accumulate in degradation mutants, suggesting not all targets of pupylation are necessarily substrates of the proteasome under steady state conditions. Four proteins implicated in *Mtb* pathogenesis, Icl (isocitrate lyase), Ino1 (inositol-1-phosphate synthase), MtrA (*Mtb* response regulator A) and PhoP (phosphate response regulator P), showed altered levels in degradation defective *Mtb*. Icl, Ino1 and MtrA accumulated in *Mtb* degradation mutants, suggesting these proteins are targeted to the proteasome. Unexpectedly, PhoP was present in wild type *Mtb* but undetectable in the degradation mutants. Taken together, these data demonstrate that pupylation regulates numerous proteins in *Mtb* and may not always lead to degradation.

## Introduction

Most individuals who are infected with *Mtb* do not develop tuberculosis. However, despite effective control of *Mtb* growth in healthy individuals, *Mtb* is rarely sterilized from the body [1]. Although numerous aspects of the immune system are responsible for slowing the growth of *Mtb*, the antimicrobial molecule nitric oxide (NO) appears to be essential for this process (reviewed in [2]). In a previous effort to identify new targets for tuberculosis therapy, *Mtb* mutants were screened for hyper-susceptibility to NO [3]. This screen identified two previously uncharacterized proteins, *Mycobacterium* proteasomal ATPase (Mpa) and proteasome accessory factor A (PafA). Proteasome protease activity encoded by *prcBA* appears to be essential for optimal in vitro growth of *Mtb* [4], [5], [6] but not the non-pathogenic relative *M. smegmatis* [7], [8].

Follow up studies revealed that PafA attaches a small protein, Pup, to at least three substrates to target them for degradation by the *Mtb* proteasome [9], [10]. Pup binds to Mpa at a ratio of 1∶6 [9], [11], [12], [13], which is thought to lead to protein unfolding for delivery into the proteasome core protease, although the latter has not been definitively demonstrated. Importantly, pupylation and proteasome function are essential for the virulence of *Mtb* for reasons that remain a mystery[3], [4], [14].

Pupylation is currently the only known post-translational protein-to-protein modification system in prokaryotes. Pup attaches to substrate lysines (K, Lys) via isopeptide bonds in a manner reminiscent of ubiquitin (Ub) and ubiquitin-like modifier (Ubl) conjugation to proteins in eukaryotes (reviewed in [15]). Proteins targeted for proteasomal degradation in eukaryotes are usually tagged with polyubiquitin chains [16], [17], [18]. Regulatory complexes associated with proteasomes recognize polyubiquitin chains, and remove and recycle Ub monomers for additional ubiquitylation reactions [19]. We do not know if Pup forms chains, or if it is recycled like Ub. Importantly, the only common feature between Ub and Pup appears to be a di-glycine motif (GG, Gly-Gly) at or near their C-termini. Ub attaches to substrate Lys via a C-terminal Gly carboxylate. Unlike Ub, Pup attaches to substrate Lys by a carboxylate group at a C-terminal glutamine (Q, Gln); however this Gln is deamidated prior to ligation [9] by deamidase of Pup (Dop) [10]. Thus the former Gln becomes a glutamate (Glu, E) and it is not known which carboxylate, the alpha or gamma, forms the isopeptide bond with substrate Lys (“GGE∼K”). Although the details of pupylation are unclear, PafA is sufficient to ligate deamidated Pup to substrates in vitro [10].

In a previous study, two-dimensional (2D) SDS-PAGE analysis showed that the proteomes of wild type and proteasome-defective *Mtb* nearly overlapped; only two proteins, FabD (malonyl CoA-acyl carrier protein acyltransferase) and PanB (3-methyl-2-oxobutanoate hydroxymethyltransferase), were conspicuously dependent on *Mtb* proteasome activity for turnover under routine culture conditions or in the presence of acidified nitrite, a source of NO [20]. Both proteins were found to be pupylated [9]. In addition, we previously determined that Mpa, the presumed proteasomal ATPase, is also a degradation substrate of the proteasome [20] and is pupylated [9]. At the time, these results led us to hypothesize that either the *Mtb* proteasome had few substrates, or that other proteins were not as robustly turned over as FabD and PanB under these conditions.

With the discovery of Pup, we were better able to comprehensively identify putative proteasomal substrates by purifying proteins that were covalently attached to Pup in *Mtb*. Here, our goals were to determine (1) if there were targets of pupylation in addition to FabD, PanB and Mpa; (2) if Pup formed chains like Ub; and (3) if pupylated proteins were necessarily proteasome targets. Based on results presented here and elsewhere [9], [20], we have now found a total of 58 pupylation targets, revealing a much broader role for Pup in protein regulation than previously expected [20]. In addition, no Pup chains were identified, suggesting poly-pupylation is not required for proteolysis or other physiological processes. Interestingly, several pupylation targets did not show increased steady state levels in degradation-defective mutants under the conditions tested. Finally, our study revealed clues that may suggest a link between proteasome-dependent proteolysis and *Mtb* pathogenesis.

## Results

### Mass Spectrometry Identifies Numerous Pupylated Proteins in *Mtb*

We used a tandem affinity purification (TAP) approach to isolate proteins covalently associated with Pup in *Mtb* under routine culture conditions (see Materials and Methods). This technique has successfully identified Ub and Ub-related modifier conjugated substrates in eukaryotes (reviewed in [21], [22], [23]). Using tandem mass spectrometry (MS/MS) we identified 604 proteins including Pup (Table S1), representing ∼15% of the total predicted proteome of *Mtb* strain H37Rv [24], [25]. Due to the high sensitivity of MS/MS it is likely that numerous proteins are not genuine pupylation targets but co-purified with true pupylated substrates. We also predict that the pupylome changes depending on the culture conditions, thus we will refer to the proteins identified under routine culture conditions as the “RCC pupylome”.

The Pup site of attachment was identified for 55 proteins, including the previously characterized FabD (Tables 1, S2) [9]. Two proteins had also been identified as pupylation targets in the non-pathogenic saprophyte *M. smegmatis* (Ino1, SodA); the same Lys in Ino1 was modified in both mycobacterial species [7] (Table 1). All linkages were GGE∼K as described for previously identified pupylation targets. There was no evidence of Pup∼Pup conjugates suggesting Pup does not form chains, however, it is possible that Pup∼Pup conjugates are present beyond the level detection, or form only under certain conditions.

**Table 1:** Proteins with known pupylation sites.

ORF:	Protein, MW:	Modified Lys:	ORF:	Protein, MW:	Modified Lys:
Rv0046c	Ino1 (40.1)	73	Rv2074c	91.3	47
Rv0073	35.8	65	Rv2115c	Mpa (67.4)	591
Rv0148	29.8	280	Rv2222c	GlnA2 (49.6)	363
Rv0242c	FabG4 (46.8)	168, 381	Rv2241	AceE (100.1)	346
Rv0357c	PurA (46.8)	292	Rv2243	FabD (30.8)	173
Rv0440	GroEL2 (56.7)	132	Rv2280	48.1	354
Rv0467	Icl (47.0)	334	Rv2419c	24.2	47
Rv0525	22.2	136	Rv2449c	44.3	127
Rv0640	RpkL (15.0)	101	Rv2477c	61.9	338
Rv0684	FusA (77.1)	307	Rv2501c	AccA1 (70.6)	321
Rv0733	Adk (20.1)	94	Rv2521	Bcp (17.0)	12, 150
Rv0814c	SseC2 (10.2)	98	Rv2606c	SnzP (31.3)	271
Rv0859	FadA (42.4)	189	Rv2624c	29.4	82
Rv0896	GltA2 (47.9)	328	Rv2676c	26.2	44
Rv1013	Pks16 (58.4)	528	Rv2737c	RecA (85.4)	762
Rv1017c	PrsA (35.5)	29	Rv2752c	59.5	502
Rv1018c	GlmU (51.6)	362	Rv2845c	ProS (63.3)	173
Rv1077	CysM2 (48.6)	428	Rv2859c	32.4	289
Rv1094	DesA2 (31.4)	145	Rv2987c	LeuD (21.8)	154
Rv1185c	FadD21 (62.8)	355	Rv3002c	IlvN (18.2)	44
Rv1295	ThrC (37.3)	151	Rv3045	AdhC (37.1)	209
Rv1308	AtpA (59.3)	489	Rv3149	NuoE (27.2)	45
Rv1315	MurA (44.0)	81	Rv3246c	MtrA (25.3)	207
Rv1392	MetK (43.0)	345	Rv3248c	SahH (54.3)	474
Rv1655	ArgD (40.9)	314	Rv3418c	GroES (10.7)	100
Rv1996	33.9	88	Rv3720	46.9	277
Rv2029c	PfkB (35.4)	283	Rv3846	SodA (23.0)	202
Rv2031c	HspX (16.2)	64, 85, 114, 132			

Of the 55 proteins with identifiable pupylation sites, most appeared to only have one site of Lys modification. Three exceptions included FabG4 (probable 3-ketoacyl-acyl carrier protein reductase) and Bcp (probable bacterioferritin co-migratory protein), each with two pupylation sites; and HspX (alpha crystallin homologue), which could have any of four of eight Lys pupylated (Table 1, S2). HspX was particularly striking as it is among the smallest proteins (16.3 kD) identified in our analysis. It was not clear if a single polypeptide could have different Lys occupied by Pup at the same time because peptides with possible pupylation sites were also found in unmodified form (Table S1).

The location of the modified Lys did not appear to favor a particular region (e.g. N- or C-termini) of the target proteins, and a conspicuous motif near the modified Lys was not identified. It is perhaps not surprising that a robust motif that indicates a favored Lys for pupylation was not found. With the exception of the eukaryotic small ubiquitin-like modifier (SUMO) family of proteins (reviewed in [26]), conserved motifs surrounding modified residues have not been observed for Ub or other Ubls.

### Pupylation Targets Include Proteins from Eight Functional Classifications

Genes in *Mtb* are assigned to one of 11 functional classifications, including stable RNAs, insertion sequences and phages [24]. All proteins identified in the RCC pupylome fall into one of seven classifications (Table 2). Notably absent were “proteins of unknown function” and “PE (proline-glutamate) and PPE (proline-proline-glutamate) proteins”; PE/PPE genes are numerous in *Mtb*, are predicted to encode secreted proteins, but their role in *Mtb* biology is poorly understood [27]. It is possible that the predicted extra-cytoplasmic membrane localization of PE/PPE proteins explains the lack of representation by this protein family in the RCC pupylome, which did not include culture supernatants or completely solubilized membranes.

**Table 2:** Classification of proteins identified in the *Mtb* pupylome.

Classification:	No. with pupylated peptide:	Percent of pupylome^1^:	Percent of predicted proteome^2^:
Intermediary metabolism	25	45	22
Conserved hypotheticals	7	13	26
Lipid metabolism	8	15	6
Information pathways	4	7	6
Cell wall, membrane-	4	7	18
associated			
Detoxification/virulence	6	11	3
Regulators	1	2	5

1 Percentage of proteins of this class identified over the total number of pupylated proteins identified by MS.

2 Percentage of this class of proteins in the total predicted *Mtb* proteome.

Members of three classifications appeared to be over-represented in the RCC pupylome when compared to their representation in the entire genome. These included “intermediary metabolism”, “lipid metabolism”, and “detoxification/virulence” (Table 2). Detoxification/virulence proteins included the chaperonins GroES, GroEL1 and GroEL2; pupylation sites were identified for GroES and GroEL2 (Tables 1, S2). We previously reported that the proteasomal ATPase Mpa is itself a target of pupylation and proteasomal degradation [9], [20], and have now identified the pupylation site (Tables 1, S2). Interestingly, several other proteins associated with proteasome function (PafA and Dop) were also identified (Table S1) [3], [10], [28]. However, we have not been able to confirm that these proteins are covalently modified with Pup.

“Cell wall processes and membrane proteins” were under-represented compared to the predicted proteome devoted to this classification. This is likely due to experimental bias and we presume much of the membrane and its components were lost during processing. Of the proteins in this class with confirmed pupylation sites, none was a predicted secreted or integral membrane protein.

### Identification of Four New Candidate Proteasome Substrates

To determine if proteins identified by TAP/MS were likely proteasome degradation substrates, we cloned 41 genes listed in Table S1, including 18 of the 27 genes encoding transcriptional regulators. None of these genes was induced (or repressed) in *mpa* or *pafA* mutant *Mtb* as determined by microarray analysis (R.A.F., K.H.D, manuscript in preparation). Each was cloned downstream of a heterologous promoter and ribosome-binding site (RBS) with a His_6_ epitope encoded at the C-terminus (see Methods; Table S3). Plasmids were used to transform wild type, *pafA* and *mpa* null mutant strains of *Mtb*, and steady state levels of putative substrates were examined by detection of the His_6_-epitope by immunoblotting. Of the 41 constructs, eight plasmids (pSYMP-Rv0681, -*phoY2*, -*hspX*, -*ino1*, -*leuD*, -*fadE24*, -*tsf*, and -*cmaA2*) resulted in undetectable recombinant protein or very few transformants of all three *Mtb* test strains, although TAP/MS had previously shown that three of the test proteins (HspX, Ino1, Tsf) were pupylated in the RCC pupylome (Table 1). The inability to detect recombinant protein may suggest the test proteins are unstable or toxic when overproduced.

**Figure 1:**
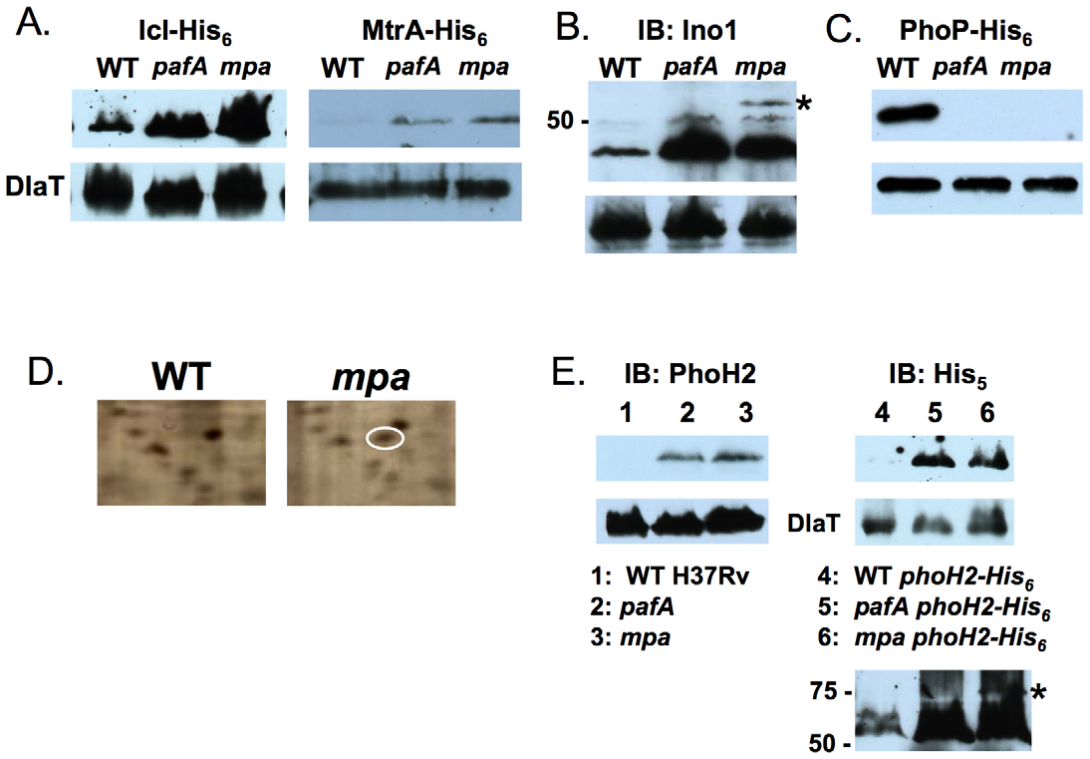
Identification of four proteasome substrate candidates. (***A***) Immunoblots using antibodies to His_5_ show that recombinant Icl-His_6_ and MtrA-His_6_ accumulate in proteasome-defective *Mtb* strains. (***B***) Polyclonal rabbit antibodies raised against Ino1-His_6_ detect accumulated endogenous Ino1 in *pafA* and *mpa* mutants compared to wild type *Mtb* H37Rv. PhoP-His_6_ is undetectable in *mpa* and *pafA* mutants. (***C***) PhoP-His_6_ is undetectable in *mpa* and *pafA* mutants. (***D***) Region of 2D SDS-PAGE gels that shows a unique spot in *mpa* mutant soluble cell lysates that was identified by MALDI-TOF MS as PhoH2. (***E***) Polyclonal rabbit antibodies raised against PhoH2-His_6_ detect endogenous PhoH2 in *pafA* and *mpa* mutants but not wild type *Mtb* H37Rv (left). Antibodies to His_5_ detect PhoH2-His_6_ synthesized in the *pafA* and *mpa* mutants but not wild type *Mtb* transformed with pSYMP-*phoH2*-His_6_ (right). Lower panel is a longer exposure of the same blot. Dihydrolipoamide acyltransferase (DlaT) is the loading control for all panels. All data are representative of at least two independent experiments from biological replicates.

Recombinant Icl (isocitrate lyase) and MtrA (*Mtb* response regulator A) accumulated in the *mpa* and *pafA* mutants, suggesting they are targets for proteasomal degradation (Fig. 1A). Both proteins had identifiable pupylation sites (Table 1). Icl is involved in the glyoxylate shunt pathway, which allows *Mtb* to utilize fatty acids or acetate as a carbon source [29] and is required for persistent infection of mice [30], [31]. MtrA is a response regulator that appears to be essential for *Mtb* growth in vitro as attempts to delete *mtrA* have been unsuccessful [32]. Over-expression of *mtrA* in *Mtb* results in slowed growth in cultured macrophages and mice, but not in broth culture [33]. The physiological relevance of artificial over-production of this transcriptional regulator remains to be determined. Although we found recombinant MtrA-His_6_ is more stable in proteasome-defective mutants, we do not know if endogenous MtrA accumulates in *mpa* or *pafA* mutants, or if pupylation affects MtrA activity.

We were interested in determining if Ino1 was a proteasome substrate since it was identified in both *M. smegmatis* [7] and *Mtb* (Table 1) as a pupylated protein. Ino1 catalyzes the first committed step of inositol synthesis and is essential for the growth and virulence of *Mtb* [34]. Because recombinant, over-produced Ino1-His_6_ was undetectable in *Mtb* we raised polyclonal antibodies to *Mtb* Ino1 to examine endogenous Ino1 levels. Like MtrA and Icl, Ino1 accumulated in the *pafA* and *mpa* mutant *Mtb* strains, and thus appears to be a proteasome substrate (Fig. 1B). The pupylated species, Pup∼Ino1, appears to accumulate in the *mpa* mutant (Fig. 1B, asterisk) similarly to Pup∼FabD [9].

In contrast to MtrA, the two-component response regulator PhoP was surprisingly less stable in the *pafA* and *mpa* mutants. In fact, PhoP-His_6_ was undetectable in the mutant strains (Fig. 1C). We speculate that PhoP interacts with another protein that is a pupylated proteasome substrate; this protein may function to target PhoP to another protease. Thus the accumulation of this hypothetical protein in a *pafA* or *mpa* mutant would reduce PhoP levels in these strains.

In addition to proteins identified by TAP/MS, we re-examined 2D-SDS-PAGE gels from a previous study [20] and identified a faint protein spot that was in lysates from an *mpa* mutant (Fig. 1D), but not wild type *Mtb*. This protein, PhoH2, was found in the RCC pupylome, is similar to PhoH from other organisms, and is predicted to bind ATP (http://genolist.pasteur.fr/TubercuList/). Although PhoH2 is annotated as a “phosphate starvation induced protein”, its actual function is unknown. Early attempts to ectopically express *phoH2* in mycobacteria resulted in toxicity to *mpa* and *pafA* mutants under normal growth conditions therefore we raised polyclonal antibodies to PhoH2-His_6_ to examine endogenous protein. PhoH2 accumulated robustly in the *pafA* and *mpa* mutants (Fig. 1E, left) in a manner similar to FabD, PanB [20], and Ino1 (Fig. 1B). We eventually succeeded in ectopic expression of *phoH2-*His_6_ using the plasmid described for the other test substrates, and observed similar results with the recombinant protein (Fig. 1E, right).

### Several Proteins Do Not Show Changes in Steady State Levels in Degradation Mutants under Standard Growth Conditions

**Figure 2:**
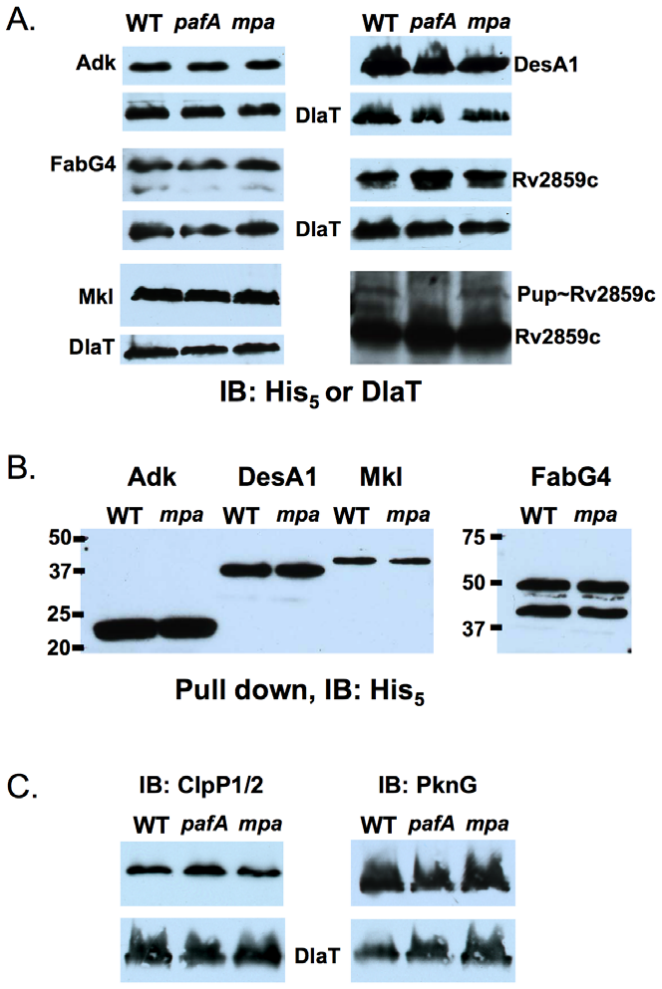
Steady state levels of several RCC pupylome proteins in wild type, *pafA* and *mpa* mutants. (***A***) Five pupylation substrates do not show increased steady state levels in proteasome-defective *Mtb*. Adk, FabG4, Mkl, DesA1 and Rv2859c were all epitope tagged with His_6_ at the C-termini. FabG4-His_6_ is predicted to have a MW of 48 kD, corresponding with the top band; a lower MW species appears in all strains tested, perhaps indicating proteolytic processing or an alternative translation initiation site after the predicted start codon. DlaT loading control is shown below each anti-His_5_ immunoblot (IB). (***B***) Purification of His_6_-tagged Adk, DesA1, Mkl, and FabG4 from wild type and *mpa Mtb* strains did not identify pupylated proteins. The upper band is the presumed full length FabG4 (without Pup). (***C***) IB analysis shows that endogenous ClpP1/2 and PknG do not accumulate in proteasome defective *Mtb* under routine culture conditions. Equivalent cell numbers from cultures grown to an optical density (OD_A580_) = 1.5 were analyzed. DlaT is the loading control for all samples.

Most of the recombinant proteins tested, including five shown to be pupylation substrates by TAP/MS (Table 1), demonstrated little to no change in steady state levels under the conditions tested (Fig. 2A, S1). Interestingly, over-exposure of immunoblots revealed what appears to be a pupylated form of Rv2859c, a possible amidotransferase (http://genolist.pasteur.fr/TubercuList/). The abundance of both the pupylated and unmodified form of Rv2859c are similar between wild type and *mpa* mutant *Mtb* (Fig. 2A, last panel), suggesting the pupylated form of this protein is not necessarily targeted for degradation under these conditions.

Although the His_6_ epitope did not previously hinder the degradation of several test substrates [20] (Fig. 1), we cannot rule out that the His_6_ tag negatively affects the stability of certain proteins in *Mtb*. Additionally, over-production of certain substrates may overwhelm the proteasome system, masking any differences in protein stability. Because we were unable to detect differences in the steady state stability of Adk (probable adenylate kinase), FabG4 (3-ketoacyl-acyl carrier protein reductase), Mkl (possible ribonucleotide-transport ATP-binding protein), or DesA1 (stearoyl-ACP destaturase) in total cell lysates, we purified the epitope-tagged proteins using affinity chromatography from wild type and *mpa* mutant *Mtb* to determine what proportion of these proteins was pupylated. Although we were able to purify the recombinant proteins from *Mtb* (Fig. 2B), we were unable to detect pupylated species of these proteins (not shown) despite MS data showing they are indeed targets of pupylation (Table 1). This suggests that these proteins are pupylated at extremely low levels or that the epitope tag on each prohibits efficient pupylation.

We also used available antibodies to examine the endogenous levels of two proteins identified in the pupylome, ClpP (caseinolytic protease) and PknG (protein kinase G). Neither protein was found to have a confirmed pupylated site therefore it was not clear if these were actual targets of pupylation. However, we chose to examine the endogenous levels of these proteins because both are predicted to be essential either for *Mtb* viability or pathogenesis [6], [35]. *Mtb* encodes two ClpP orthologues, ClpP1 and ClpP2, which appear to be encoded in an operon. TAP/MS identified both ClpP1 and ClpP2 (Table S1). ClpP proteases are similar to proteasomes in that they form chambers and require ATP for regulated proteolysis (reviewed in [36]). Unlike proteasomes, which are only found in bacteria of the class Actinomycetes, ClpP proteases are found in all bacteria sequenced to date. Polyclonal antibodies to ClpP1/2 detected a single band in *Mtb* lysates (Fig. 2C, left). ClpP1 and ClpP2 are similar in molecular weight (22 and 24 kD, respectively) therefore we could not determine which of the two proteins, if not both, was detected. Nonetheless, we did not observe a difference in the steady state levels of this protein among the wild type and mutant *Mtb* strains. We also examined the steady state levels of PknG in wild type and proteasome defective *Mtb* strains. PknG is a serine-threonine kinase that is required for *Mtb* survival in cultured macrophages [35]. Similar to ClpP1/2, we did not detect a difference in steady state PknG levels among the *Mtb* strains (Fig. 2C, right).

### Enzymes of the Fatty Acid Synthase II (FASII) Pathway Are Differentially Regulated by the Pup-Proteasome System

**Figure 3:**
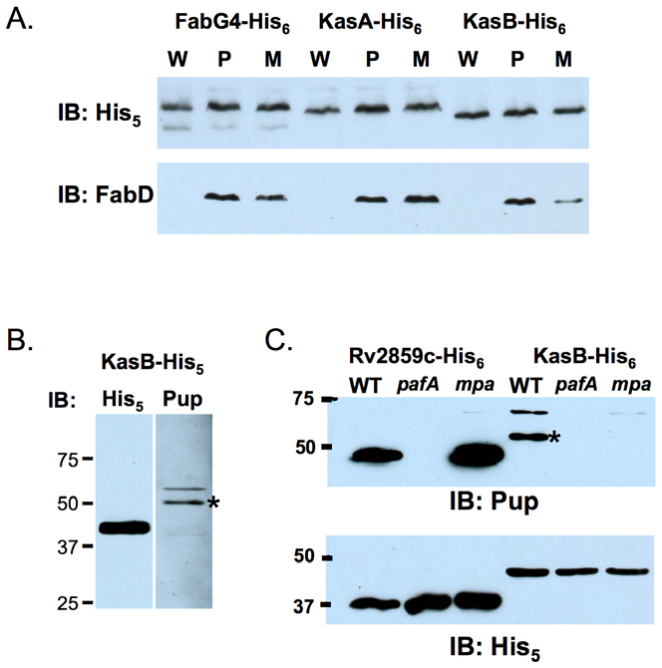
Fatty acid synthase (FASII) enzymes are differentially regulated by the Pup-proteasome system. (***A***) Anti-His_5_ immunoblot of FASII pathway enzymes that are confirmed or putative pupylation targets have differing steady state levels in proteasome-defective *Mtb*. The same KasA and KasB samples are also shown with the loading controls in Fig. S1. KasA (MW = 43 kD) migrates more slowly than KasB (MW 46 kD); similar observations were previously reported [54]. The membrane was stripped and endogenous FabD was examined with FabD antibodies. (***B***) KasB-His_6_ is positive for pupylation. Pup antibodies show a specific Pup-reactive protein (*) from KasB-His_6_ purified from *M. smegmatis*. The protein above the indicated band (*) is non-specific and recognized in all samples from *M. smegmatis*. All data are representative of at least two independent experiments from biological replicates. (***C***) Pup∼KasB (*) is undetectable in the *pafA* and *mpa* mutants. His_6_-tagged Rv2859c and KasB were purified from *Mtb* and analyzed by immunoblotting with antibodies to Pup, stripped, and then re-examined with antibodies to His_5_. Due to the unexpected result for the *mpa* mutant, we confirmed that the *mpa* strain was pupylation proficient by anti-Pup immunoblotting (not shown). The higher MW species appears to specifically co-purify with Pup∼KasB and is recognized by the Pup antibody. Equivalent cell numbers were analyzed and all experiments are representative of two replicates.

Every protein encoded in the fatty acid synthase II (FASII) elongation cycle operon (FabD, AcpM, KasA, KasB, AccD6) was identified in the pupylome (Table S1), suggesting that nearly all proteins in this pathway are targets of pupylation, or that unpupylated FASII enzymes co-purified with pupylated proteins. As discussed earlier, FabG4, which is part of the FASII pathway but genetically unlinked, was shown to be pupylated on either of two Lys (Table 1), but not a proteasome substrate under normal culture conditions (Fig. 2A). FabD is encoded by the first gene in the FASII operon and is a robust degradation substrate [9], [20]. Unlike FabD, KasA and KasB (beta-ketoacyl-ACP synthases 1 and 2, respectively) steady state levels were not noticeably different between the wild type and *mpa* and *pafA* strains (Fig. 3A, S1). We purified KasA-His_6_ and KasB-His_6_ from *M. smegmatis* for anti-Pup immunoblot analysis and observed a specific Pup-reactive protein in the KasB pull down (Fig. 3B), but not in the KasA sample (not shown). We then determined if Pup∼KasB was present in *Mtb*. As a positive control for the pull down of pupylated substrate, we purified Rv2859c-His_6_, which has a known pupylation site and for which a pupylated species could be detected in total cell lysates (Fig. 2A). As expected, we detected Pup∼Rv2859c in the wild type and *mpa Mtb* strains, but not in the *pafA* (pupylation-deficient) mutant (Fig. 3C). To our surprise, although Pup∼KasB was present in wild type *Mtb*, it was not detected in either the *pafA* or the *mpa* mutant (Fig. 3C). This suggested that a defect in Mpa function and/or proteolysis by the proteasome results in either abrogated pupylation, or possibly increased “de-pupylation” of KasB. Taken together, it appears that the Pup-proteasome pathway differentially affects enzymes of the FASII pathway.

## Discussion

In this study we identified the pupylation sites of 55 proteins in *Mtb*, including the previously identified FabD and Mpa [9]. Using the same pull-down techniques for identifying Pup∼FabD and Pup∼PanB [9], we determined that KasB is also pupylated (Fig. 3). We presume the degradation substrate PhoH2 is also pupylated, bringing our current pupylome total to 58 proteins. Three proteins have more than one pupylation site, but we did not identify Pup chains. To date, we have identified seven likely proteasome substrates, including FabD, PanB, Mpa [20]; and the newly identified PhoH2, Icl, MtrA, and Ino1.

Five of proteins with confirmed pupylation sites were not differentially turned over between wild type and proteasome-defective *Mtb* (Fig. 2). It is notable that three of the six known degradation substrates, FabD, PanB and PhoH2, were identified by comparing the steady state proteomes of wild type and degradation defective *Mtb* by 2D-SDS-PAGE; the two proteomes appeared nearly identical [20]. These data are also consistent with the observation that numerous anti-Pup reactive proteins are present in *Mtb*, and the abundance of most of these proteins does not change in a degradation mutant [9]. However, the lack of accumulation of pupylated substrates in a degradation-defective strain might be due to negative feedback regulation, where a defect in proteolysis results in reduced pupylation.

There are several possible explanations for why recombinant pupylation targets are not proteasome substrates. First, the reasons may be technical; it is possible that over-production of certain proteins overwhelms the Pup-proteasome system, masking any observable differences in proteolysis of these substrates. It is also possible that the His_6_-epitope prevents pupylation or degradation of certain proteins. Additionally, not all proteins may be pupylated to the same degree; for all of the test substrates, we do not know what percentage of each protein is pupylated. We also predict that certain proteins are differentially pupylated (and degraded) under specific conditions. Other proteins may be required to deliver a pupylated substrate to the proteasome, and these co-factors may not be expressed under the conditions tested. Finally, not all pupylated proteins may be targets of proteasomal degradation, a potentially exciting observation. Ub and related modifiers have numerous activities in eukaryotes [37], controlling processes from signal transduction to regulating enzyme activity, in addition to targeting proteins for degradation, therefore, we speculate that Pup has widespread, degradation-independent importance in bacterial physiology. How and why certain proteins are more efficiently targeted for pupylation and/or proteolysis are questions currently under investigation.

It is striking that the steady state level of one protein, FabD, is strongly regulated by pupylation while other confirmed (KasB, FabG4) and presumed (KasA) pupylated proteins in the same pathway are not rapidly turned over under the same conditions. Perhaps most intriguing is the observation that not only does Pup∼KasB not accumulate in an *mpa* mutant, but Pup∼KasB appears to be present at extremely low to undetectable levels in this strain (Fig. 3C). We do not yet understand the significance of this result, but we speculate that the absence of Mpa either reduces the pupylation of KasB, or increases the removal of Pup. It is also possible that Mpa protects Pup∼KasB from proteolysis by another protease.

Because proteasome function is essential for *Mtb* pathogenesis [3], [4], [14], [20], [38] we predicted the identification of proteasomal degradation substrates may give clues to link proteasome-dependent proteolysis and virulence. The inability to turn over potentially hundreds (or even a handful) of proteins could greatly compromise bacterial survival when adapting to a new environment, such as the inside of an activated macrophage. It is unknown what effect, if any, the accumulation of metabolic enzymes, such as FabD, PanB, Ino1, Icl or PhoH2, has during an infection. Our data show that several virulence-associated proteins are regulated by proteasomal degradation. The transcriptional response regulator MtrA is pupylated and appears to be a proteasome substrate. Over-expression of MtrA attenuates *Mtb* in macrophages and mice [33] thus the accumulation of MtrA in a proteasome mutant could potentially contribute to the weakened virulence of proteasome-defective *Mtb*. In contrast to MtrA, the global regulator PhoP was *less* stable in the *pafA* and *mpa* mutants. PhoP is essential for the virulence of numerous pathogens, including *Mtb* [39], [40], [41], [42], [43]. PhoP is required for *Mtb* growth in both macrophages and mice [39], [42]. A point mutation in the DNA binding domain of PhoP in the avirulent *Mtb* strain H37Ra is thought to account for part of its attenuation compared to the virulent parent strain H37Rv [44], [45]. The cause of attenuation of a *phoP Mtb* mutant is unclear, although PhoP appears to impact numerous aspects of *Mtb* biology, including lipid metabolism [39], [41], [46], early and enduring hypoxic responses [46], respiration, and virulence gene expression [46], [47]. The lack of PhoP in the *pafA* and *mpa* mutants thus suggests an important role for proteasome activity on *Mtb* pathogenesis. A long-term goal will be to determine why PhoP stability is decreased in proteasome-defective *Mtb*.

Although our study has shed light on several aspects of the Pup-proteasome system, numerous additional questions have been raised. For example, how does a single ligase, PafA, target at least 58 or potentially hundreds of proteins for pupylation in *Mtb*? In eukaryotes, Ub ligases are numerous in order to provide specificity to ubiquitylation; how is specificity imparted in the Pup system? What signals in addition to Pup are required to target proteins for proteolysis? Does Pup play non-degradative roles in the cell? There is little doubt that considerable effort will be needed to determine how so many processes targeted by pupylation impact the physiology of *Mtb*.

## Materials and Methods

### Bacterial Strains, Plasmids, Media

All strains, primers and plasmids are listed in Table S3. For cloning and transformations we used *E. coli* DH5α (Gibco, BRL). For protein synthesis we used *E. coli* ER2566 [48]. All *Mtb* work was performed in a Biosafety Level 3 (BSL3) suite. *Mtb* H37Rv was used for all *Mtb* studies. *mpa* and *pafA* mutants are described elsewhere [3]. *E. coli* strains were grown in Luria Bertani (LB) Broth (Difco). *Mtb* strains were grown on 7H11 Middlebrook agar supplemented with oleic acid, dextrose and catalase (Middlebrook OADC, Difco) or in Middlebrook 7H9 broth (Difco) supplemented with 0.2% glycerol, 0.05% Tween-80, 0.5% bovine serum albumin, 0.2% dextrose and 0.085% sodium chloride. *Mtb* cultures were grown without shaking in 75 cm^2^ vented flasks in humidified incubators with or without 5% CO_2_. For *Mtb*, kanamycin and hygromycin were used as needed at 50 µg/ml each. For *E. coli*, kanamycin and hygromycin were used at 100 and 150 µg/ml, respectively. Isopropyl-β-D-thiogalactopyranoside (IPTG) was used at a final concentration of 1 mM.

For the identification of pupylated targets in *Mtb*, a tandem affinity tagged version of Pup (pMN-His_6_-Strep-Pup), was constructed. *Mtb phoH2*, *ino1*, and *fabD* were cloned into pET24b(+) for the purification of His_6_-FabD, Ino1-His_6_, and PhoH2-His_6_ for antibody production (see Table S3 for primers used for cloning).

For over-production of putative pupylation targets in *Mtb* we used a modified form of pMN402, which has green fluorescent protein (*gfp*) encoded downstream of a mycobacterial heat shock protein promoter (*hsp60p*) and a consensus bacterial RBS [49]. Transformation of pMN402-derivatives that had *gfp* replaced with genes of interest often resulted in toxicity to *Mtb*, most likely due to strong over-expression of the cloned gene from the *hsp60p* and robust translation from the consensus RBS. To reduce protein synthesis by this plasmid, a short coding sequence ending with a consensus RBS and a start codon was cloned downstream of the *gfp* RBS and start codon. Genes of interest were cloned starting with the second start codon. We predicted the final plasmid expressed a transcript encoding a short peptide followed by the test substrate (all final constructs were designated “pSYMP”). As a result few of the test substrates were toxic to *Mtb*. Genes were amplified with primers that encoded a His_6_ epitope at the 3′ termini.

All primers were from Invitrogen. All plasmids were sequenced by GENEWIZ (South Plainfield, NJ) to confirm the veracity of the cloned sequences. Transformation was carried out using standard techniques for *E. coli* [50]. Wild type, *pafA* and *mpa* mutant strains of *Mtb* were transformed with test plasmids using electroporation [51]. *Mtb* transformants arose after 2–3 weeks and three colonies from each transformation were picked into 200 µl 7H9 broth with antibiotics in 96 well plates. After one week at 37°C, these starter cultures were inoculated into 5 ml 7H9 in vented 25 cm^2^ flasks (Corning), in duplicate, for further growth. After another week cultures were frozen or used for immunoblot analysis.

### Purification of the RCC Pupylated Substrates

0.5 l WT *Mtb* containing pMN-His_6_-Strep-Pup was grown to an OD_580_ of ∼1.0. Bacteria were collected by centrifugation, resuspended in 14 ml of denaturing lysis buffer B (The QiaExpressionist manual, Qiagen) and 1 ml aliquots of cells were transferred to bead beating tubes each with 250 µl of zirconia silica beads (BioSpec Products). Cells were lysed by bead beating in a BioSpec Mini Bead Beater. Samples were clarified by centrifugation and were then passed through a 0.2 µ filter. The lysate was incubated with 1.5 ml of Ni-NTA agarose (Qiagen) for 2 h at 4°C with agitation. The agarose was collected in a polypropylene column and washed with denaturing wash buffer. The matrix was then washed with 1 column volume plus 10 ml of native wash buffer. Proteins were eluted with 3.5 ml native elution buffer. The eluate was incubated with 1 ml of Strep-Tactin Superflow (Qiagen) for 2 h at 4°C with agitation. The column was washed three times with 5 ml of Strep wash buffer (50 mM NaH_2_PO_4_/300 mM NaCl, pH 8.0) and proteins were eluted in six 500 µl fractions with Strep elution buffer (50 mM NaH_2_PO_4_/300 mM NaCl/2.5 mM desthiobiotin, pH 8.0). 40 µl of elution two was visualized on a 12% SDS-PAGE gel stained with Biosafe Coomassie Blue (Bio-Rad). The entire lane was excised for tandem MS/MS analysis.

### Proteomic Analysis

For identification of proteins pulled down by TAP we used in-gel proteolysis and LC-MS/MS analysis. The gel lane was divided into six bands, each of which was cut into ∼1 mm cubes and transferred into 1.5 ml Eppendorf tubes. The procedure for in-gel digestion was as described previously [52]. Briefly, the proteins were digested overnight with trypsin at 37°C in 50 mM ammonium bicarbonate. Peptides were extracted with 50% acetonitrile, 5% formic acid, dried by vacuum centrifugation and desalted using handmade StageTips containing C18 resin [53]. Peptides were eluted with 50% acetonitrile, 5% formic acid into glass inserts, and dried in a Speed-Vac.

The samples were analyzed on an LTQ Orbitrap XL hybrid mass spectrometer (ThermoFisher) coupled to an Agilent 1100 series binary pump. Dried peptides were reconstituted in 8 µl 5% acetonitrile, 5% formic acid of which 4 µl was loaded onto a hand-pulled fused silica microcapillary (125 µm×15 cm, packed with Magic C18AQ, Michrom Bioresources) using a Famos autosampler (LC Packings). Loaded peptides were separated across a 45 m linear gradient of 10–37% solvent B (0.125% formic acid, 99.875% acetonitrile). Solvent A comprised 0.125% formic acid, 3% acetonitrile. Data were collected in a data-dependent mode using the TOP10 strategy where one full high resolution MS scan was acquired in the orbitrap followed by 10 MS/MS scans in the linear ion trap from the top 10 most intense ions.

RAW files were converted to mzXML files using the program ReAdW (http://sashimi.sourceforge.net/software_glossolalia.html). MS/MS spectra were searched using the SEQUEST search algorithm (version 27, revision 12) against an *Mtb* protein database [24] using a mass tolerance of 50 ppm and a static modification of 57.02146 Da (carboxyamidomethylation). The search parameters for post-translational modifications comprised dynamic modifications of 243.085522 Da on Lys (GGE) and 15.99491 Da on methionine (oxidation). Partial Trypsin was specified with a maximum of two missed cleavages.

Protein hits were filtered at the peptide level to contain less than 0.22% false positives, estimated by the number of decoy hits using in-house software and filtering based on dCn, XCorr, ppm, charge state and peptides per protein. The protein false positive rate was estimated to be 1.47%.

PhoH2 was identified by the Columbia Proteomics Core Facility from spots found in 2D gels prepared by kendricklabs.com [20].

### Protein Purification and Immunoblot Analysis

FabD-His_6_ and Ino1-His_6_ were purified under native conditions; PhoH2-His_6_ was purified under denaturing conditions as described elsewhere (The QiaExpressionist). PhoH2-His_6_ was excised from a 10% SDS-PAGE gel. Antibodies to PhoH2 and FabD were made by Sigma-Genosys; antibodies to Ino1 were made by Covance. All rabbit antibodies were diluted in 1% skim milk in TBST (25 mM Tris-HCl, pH 7.4/125 mM NaCl/0.05% Tween 20). Secondary antibodies (GE-Healthcare) were diluted in 1% milk/TBST. To examine epitope tagged protein levels each strain was grown under standard conditions until stationary phase (optical density at A_580_ between 1–2; all samples were OD matched). All cultures were processed exactly as described elsewhere [38]. For immunoblot analysis, cell lysates were separated by 10 or 12% SDS-PAGE, transferred to nitrocellulose membranes and blocked in 3% bovine serum albumin/TBST and incubated with monoclonal Penta-His (His_5_) antibodies (Qiagen) or secondary antibodies (GE-Healthcare) in 1% milk/TBST.

To test is specific proteins were pupylated in mycobacteria, the gene encoding the protein of interest was expressed from pSYMP in *M. smegmatis* or *Mtb*. *M. smegmatis* was grown in 250 ml 7H9 and processed as described in the QiaExpressionist manual for purification under denaturing conditions. For *Mtb*, equivalent cell numbers based on A_580_ readings (typically 17 optical density units) were collected by centrifugation, washed with PBS/0.05% Tween-80, and resuspended in 3 ml native lysis buffer (QiaExpressionist). Bacteria were lysed by bead beating and unbroken cells were removed by centrifugation. The supernatants were then filtered through a 0.22 µ nylon filter and incubated with 30 µl NiNTA agarose for 2 h at 4°C on a rotator. The agarose was washed four times with native wash buffer then eluted in 60 µl native elution buffer. 20 µl 4 5 protein sample buffer was added to eluates and samples were analyzed by immunoblotting with antibodies to His_5_.

## Supporting Information

Figure S1 Steady state levels of many RCC pupylome proteins do not differ between wild type, *pafA* and *mpa* mutants. Immunoblots of equivalent cell numbers from cultures grown to an optical density (ODA580) = 1.5 were analyzed. All blots were stripped with 0.2 N sodium hydroxide and incubated with antibodies to DlaT, the loading control (lower panel for each blot).
(1.44 MB PDF)

Table S1 Peptide profile of the pupylome.
(1.33 MB XLS)

Table S2 *Mtb* peptides with pupylation sites.
(0.07 MB DOC)

Table S3 Bacterial strains, plasmids and primers used in this work.
(0.05 MB DOC)
