# Correction: Prokaryotic Ubiquitin-Like Protein (Pup) Proteome of *Mycobacterium tuberculosis*

**DOI:** 10.1371/annotation/bf95b2c0-4085-417b-a2b2-7a85ffe77a9e

**Published:** 2010-07-29

**Authors:** Richard A. Festa, Fiona McAllister, Michael J. Pearce, Julian Mintseris, Kristin E. Burns, Steven P. Gygi, K. Heran Darwin

There was a typographic error in the title of this article. The correct title is: Prokaryotic Ubiquitin-Like Protein (Pup) Proteome of Mycobacterium tuberculosis. The correct citation is: Festa RA, McAllister F, Pearce MJ, Mintseris J, Burns KE, et al. (2010) Prokaryotic Ubiquitin-Like Protein (Pup) Proteome of Mycobacterium tuberculosis. PLoS ONE 5(1): e8589. doi:10.1371/journal.pone.0008589 

