# Supplementary figures and images for: Prokayrotic Ubiquitin-Like Protein (Pup) Proteome of Mycobacterium tuberculosis
Source: PLoS One. 2010 Jan 6;5(1):e8589. doi: 10.1371/journal.pone.0008589 (PMC2797603; doi:10.1371/journal.pone.0008589)

Festa et al Fig. S1

| Lane: | Strain:     |
|-------|-------------|
| W     | WT          |
| P     | <i>pafA</i> |
| M     | <i>mpa</i>  |

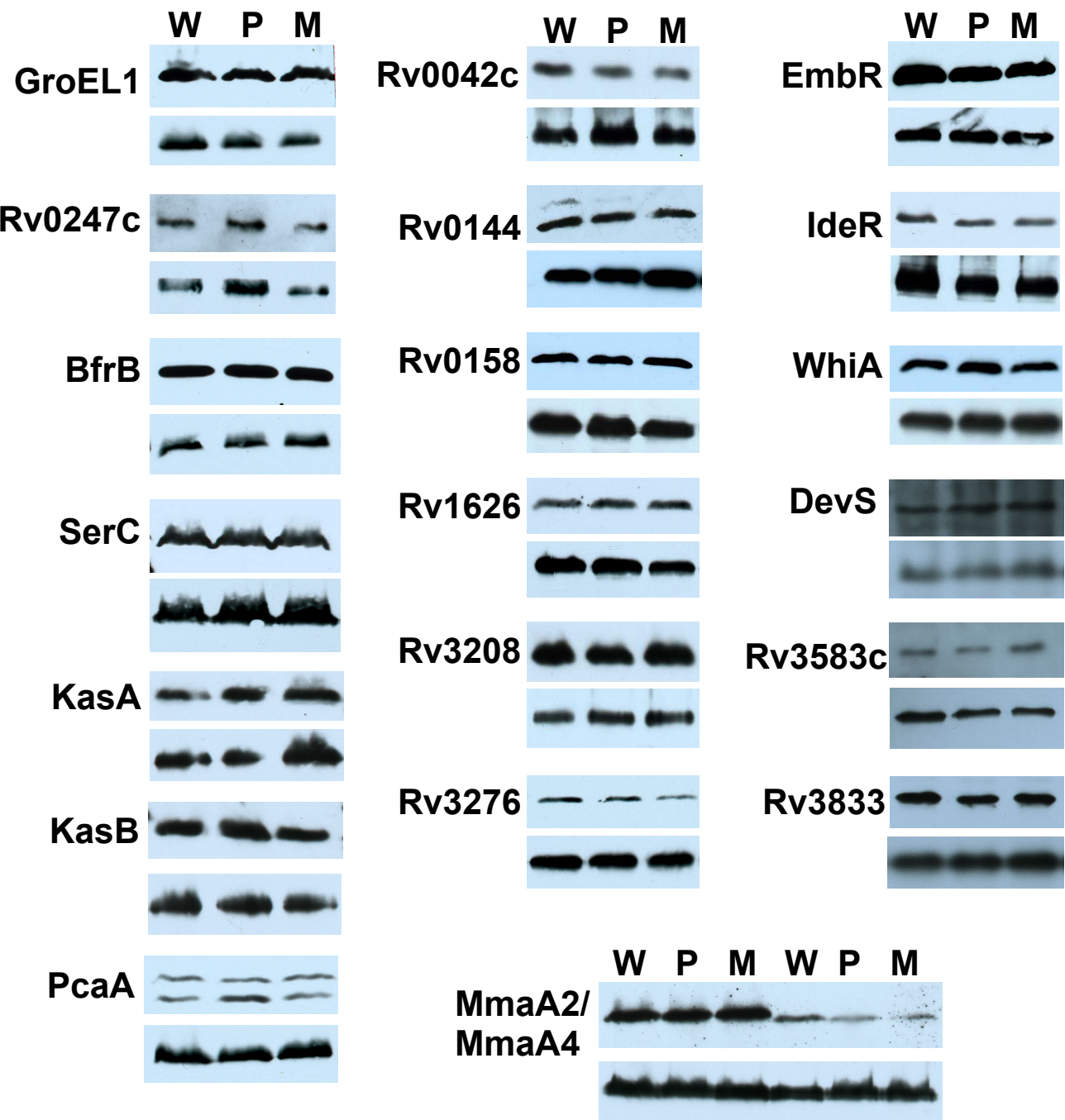

Supplement: Figure S1 — Steady state levels of many RCC pupylome proteins do not differ between wild type, pafA and mpa mutants. Immunoblots of equivalent cell numbers from cultures grown to an optical density (ODA580) = 1.5 were analyzed. All blots were stripped with 0.2 N sodium hydroxide and incubated with antibodies to DlaT, the loading control (lower panel for each blot). (1.44 MB PDF) [file pone.0008589.s001.pdf]
