# Supplementary material for: Prokayrotic Ubiquitin-Like Protein (Pup) Proteome of Mycobacterium tuberculosis
Source: PLoS One. 2010 Jan 6;5(1):e8589. doi: 10.1371/journal.pone.0008589 (PMC2797603; doi:10.1371/journal.pone.0008589)
Supplement: Table S2 — Mtb peptides with pupylation sites. (0.07 MB DOC) [file pone.0008589.s003.doc]

**Table S2. *Mtb* peptides with pupylation sites.**

| Rv0046c | Ino1 | K.FVAAFDVDAK#K.V | Rv2222c | GlnA2 | R.GVEK#GYVLGPQAEDNVWDLTPEER.R |
| --- | --- | --- | --- | --- | --- |
| Rv0073 |  | K.SGAIK#FDEVDITTLQGAELANYR.R | Rv2241 | AceE | R.TK#ALVENMSDQDIWNLKR.G |
| Rv0148 |  | R.WAEITDLSGAK#IAGFK.L | Rv2243 | FabD | R.LTALEK#LAEDPPAK.A |
| **Rv0242c** | FabG4 | K.AGMIGITQALAPGLAAK#GITINAVAPGFIETQ.M | Rv2280 |  | R.ASM*PK#FLSTAR.G |
| **Rv0242c** | FabG4 | R.GATTALVYLSPDAK#PAATGLESTM*R.F | Rv2419c |  | R.TQAVAAAEVLGK#R.Q |
| Rv0357c | PurA | R.VGSGPFPTELFDEHGEYLSK#TGR.E | Rv2449c |  | R.NSIDLYHK#QAADTGAR.I |
| Rv0440 | GroEL2 | K.VTETLLK#GAK.E | Rv2477c |  | R.ALIK#DLSFSLPR.N |
| Rv0467 | Icl | K.FQK#ELAAM*GFK.F | Rv2501c | AccA1 | R.VGAGEK#LGFAQNDIELR.G |
| Rv0525 |  | R.MTTAVDK#AR.V | **Rv2521** | Bcp | K.ATGHVAK#LR.R |
| Rv0640 | RpkL | K.VAK#VTWDQVR.E | **Rv2521** | Bcp | R.LTPGDK#APAFTLPDADGNNVSLADYR.G |
| Rv0684 | FusA | R.K#ATTDEPFAALAFK.I | Rv2606c | SnzP | K.ATTFFDDPDVLAK#VSR.G |
| Rv0733 | Adk | R.SVEQAK#ALHEM*LER.R | Rv2624c |  | K.LVK#IETDIPR.G |
| Rv0814c | SseC2 | R.ALSAAGNGDAVVQPSGAGIHEVDVK#IT.- | Rv2676c |  | R.DAIIDDASTFFK#QQEER.G |
| Rv0859 | FadA | K.AAEAWSGGYFAK#SVVPVR.D | Rv2737c | RecA | R.NFLVENADVADEIEK#K.I |
| Rv0896 | GltA2 | K.EQADK#ILAK.L | Rv2752c |  | R.GFSEDPK#ALEPAVR.K |
| Rv1013 | Pks16 | R.NVVVLGPGTIPK#TPSGK.L | Rv2845c | ProS | K.DSYSFDIDAAGLK#AAYHAHR.E |
| Rv1017c | PrsA | R.AHPELAEQVAK#ELDVHVTSQDAR.E | Rv2859c |  | R.LFK#ALVDAASGYAGR.Q |
| Rv1018c | GlmU | G.ADGKLGAFVEVK#.N | Rv2987c | LeuD | R.IITAATVVLPFK#IDDHSAWR.L |
| Rv1077 | CysM2 | R.M*IGAGELVSAAGK#ALR.D | Rv3002c | IlvN | R.RGFNIESLAVGATECK#DR.S |
| Rv1094 | DesA2 | R.AEK#YTQVETLVY.M | Rv3045 | AdhC | K.LGAAM*GADVTVLSQSLK#K.M |
| Rv1185c | FadD21 | R.NSGDKPEVVYFEPDK#LSTGSANR.C | Rv3149 | NuoE | R.LEVDAK#EIIGR.Y |
| Rv1295 | ThrC | R.K#M*AADFPTISLVNSVNPVR.I | Rv3246c | MtrA | R.AKVEK#DPENPTVVLTVR.G |
| Rv1308 | AtpA | K.LTEEAADK#LTEVIK.N | Rv3248c | SahH | K.LTK#EQAEYLGVDVEGPYKPDHYR.Y |
| Rv1315 | MurA | R.ITAPDEPK#YDADFAAVR.Q | Rv3418c | GroES | R.DVLAVVSK#.- |
| Rv1392 | MetK | K.AAPVGLFVETFGTETEDPVK#IEK.A | Rv3720 |  | R.VIGATLSAEQAK#WGQK.A |
| Rv1655 | ArgD | R.AEVLGK#SLR.H | Rv3846 | SodA | R.YAAATSQTK#GLIFG.- |
| Rv1996 |  | R.EIVEHSYLVAQAHQIVEQAHK#VALEASSSGR.A |  | | |
| Rv2029c | PfkB | R.GWSLIK#SVR.L |  | | |
| **Rv2031c** | HspX | D.PDK#DVDIMVR.D |  | | |
| **Rv2031c** | HspX | K.GILTVSVAVSEGK#PTEK.H |  | | |
| **Rv2031c** | HspX | R.AELPGVDPDK#DVDIMVR.D |  | | |
| **Rv2031c** | HspX | R.TEQK#DFDGR.S |  | | |
| **Rv2031c** | HspX | R.TVSLPVGADEDDIK#ATYDK.G |  | | |
| Rv2074 |  | R.ADNSPHVVAVGFTFDPK#THIAR.V |  | | |
| Rv2115c | Mpa | R.TLVTGK#SSSASR.A |  | | |

#Indicates modified Lys; * indicates oxidized methionine.
