# Supplementary material for: Prokayrotic Ubiquitin-Like Protein (Pup) Proteome of Mycobacterium tuberculosis
Source: PLoS One. 2010 Jan 6;5(1):e8589. doi: 10.1371/journal.pone.0008589 (PMC2797603; doi:10.1371/journal.pone.0008589)
Supplement: Table S3 — Bacterial strains, plasmids and primers used in this work. (0.05 MB DOC) [file pone.0008589.s004.doc]

**Table S3.** Bacterial strains, plasmids and primers used in this work**.**

***E. coli:* Relevant genotype: Source or reference:**

DH5α F-, p80d*lacZ*ΔM15 Δ(*lacZYA-argF*)U169 *deoR recA1 endA1*  Gibco, BRL

*hsdR17* (r_k_-m_k_+) *phoA supE44* λ- *thi-1 gyrA96 relA1*

ER2566 F- λ- *fhuA2* [lon] *ompT lacZ::T7 geneI gal sulA11* {Chong, 1994 #200}

Δ(*mcrC-mrr*)114::IS10 R(*mcr-73*::miniTn10)2

R(*zgb-210*::Tn10)1 (Tets) *endA1* [dcm]

***M. smegmatis:***

mc^2^155 wild type {Snapper, 1990 #477}

***M. tuberculosis:***

H37Rv wild type American Type Culture Collection 25618 ATCC

MHD2 Kan^r^; *pafA*::ΦMycoMarT7 (Kan^r^) {Darwin, 2003 #162}

MHD5 Kan^r^; *mpa*::ΦMycoMarT7 (Kan^r^) {Darwin, 2003 #162}

**Plasmids and primers**

**(sequences are 5’ to 3’):**

pET24b(+) Kan^r^; for production of C-terminal His_6_ epitope-tagged protein Novagen

pET24b(+)-*phoH2* Kan^r^; for expression of *phoH2*-his_6_ This study.

NdephoH2f2 ggaattccatatgaccgatacccgcacgtacgtgctc

phoH2-BamHI-r1 aaggatcctcagtggtggtggtggtggtggcgcggcc

cggtgatctcctcgag

pET24b(+)-*fabD* Kan^r^; His_6_ epitope cloned upstream of *Mtb* *fabD*. This study.

His-FabD-Nde-F GGAATTCcatatgcaccaccaccaccaccacatgattg

cgttgctcgcacccgga

FabD_STOP_HIII_R CCCAAGCTT TTATAGGTTTGCCAGCTCGTCCAGGTC

pET24b(+)-*ino1* Kan^r^; for expression of *ino1*-his_6_ This study.

Rvinofor cgcccatatgagtgagcaccagt g

Rvinorev caacggcggccgcaccgatgatgaactc

pMN402 Hyg^r^; shuttle plasmid with *gfp* under the control of the {Scholz, 2000 #319}

BCG *hsp60* promoter

pMN-His-Strep-Pup Hyg^r^; for purification of the pupylome from *Mtb*. This study.

HisStrep_F TATGGCACACCACCATCACCATCACGCACTCTGGAGCCA

CCCGCAGTTCGAAAAAGGTAC

HisStrep_R CTTTTTCGAACT GCGGGTGGCTCCAGAGTGCGTGATGGTG

ATGGTGGTGTGCCA

kpn_prcC_F ggGGTACCgcgcaagagcagaccaa

pst_prcC_R aaaaCTGCAGtcactgtccgcccttttggacgt

***pSYMP plasmids and primers:***

All carry hygromycin-resistance, have the cloned gene under control of the *hsp60p* and encode a C-terminal His_6_ epitope.

pSYMP-*adk* adk_Nde_F GGGAATTCCATATGagagttttgttgctgggaccg

adk_HIII_R CCCAAGCTTtcagtggtggtggtggtggtgctttcccagagcccgcaa

pSYMP-*hspX* hspX_Nde_F GGGAATTCCATATGgccaccacccttcccgttc

hspX_HIII_R CCCAAGCTTtcagtggtggtggtggtggtggttggtggaccggatctgaatgt

pSYMP-*desA1* desA1_Nde_F GGGAATTCCATATGtcagccaagctgaccgacct

desA1_RV_R CCGGATATCtcagtggtggtggtggtggtgacgacggctcatcgccagtt

pSYMP-*fabG4* fabG4_Nde_F GGGAATTCCATATGgctcccaagcgttcgtccgat

fabG4_HIII_R CCCAAGCTTtcagtggtggtggtggtggtgcgcgccgatcatggcctg

pSYMP-*ino1* ino1_Nde_F GGGAATTCCATATGagtgagcaccagtcgttacc

ino1_HIII_R CCCAAGCTTtcagtggtggtggtggtggtgaccgatgatgaactcttcgag

pSYMP-*kasA* kasA_Ase_F GGGAATTCATTAATGagtcagccttccaccgct

kasA_HIII_R CCCAAGCTTtcagtggtggtggtggtggtggtaacgcccgaaggcaag

pSYMP-*kasB* kasB_Nde_F GGGAATTCCATATGggggtccccccgcttgcg

kasB_HIII_R CCCAAGCTTtcagtggtggtggtggtggtggtaccgtccgaaggcgattgccac

pSYMP-*mkl* mkl_Nde_F GGGAATTCCATATGcgatacagtgactcataccacacaacg

mkl_HIII_R CCCAAGCTTtcagtggtggtggtggtggtgctggccgatttcgtgcacc

pSYMP-*mmaA2* mmaA2_Nde_F GGGAATTCCATATGgtcaacgacctaacgccgcac

mmaA2_HIII_R CCCAAGCTTtcagtggtggtggtggtggtgcttcgccagcgtgaactggttg

pSYMP-*mmaA4* mmaA4_Nde_F GGGAATTCCATATGacgagaatggccgagaaaccgatt

mmaA4_HIII_R CCCAAGCTTtcagtggtggtggtggtggtgggccgcggcacccggcttg

pSYMP-*pcaA* pcaA_Nde_F GGGAATTCCATATGtccgtgcagctcacgccgc

pcaA_HIII_R CCCAAGCTTtcagtggtggtggtggtggtgcttttccagtgtgaactggtcgacgtc

pSYMP-*leuD* leuD_Nde_F GGGAATTCCATATGgaagcctttcacacccactctgg

leuD_HIII_R CCCAAGCTTtcagtggtggtggtggtggtggggggcgggtagagtgcg

pSYMP-*fadE24* fadE24_Nde_F GGGAATTCCATATGaccaacaccacctctgctg

fadE24_HIII_R CCCAAGCTTtcagtggtggtggtggtggtggatgacaacaacgccctcg

pSYMP-*tsf* tsf_Nde_F GGGAATTCCATATGgcgaacttcactgccgctg

tsf_HIII_R CCCAAGCTTtcagtggtggtggtggtggtgagcctggcccacctcgaa

pSYMP-*bfrB* bfrB_Nde_F GGGAATTCCATATGacagaatacgaagggcctaagacaaaattc

bfrB_HIII_R CCCAAGCTTtcagtggtggtggtggtggtggaggcggcccccggcagc

pSYMP-*clpP2* clpP2_Nde_F GGGAATTCCATATGaattcccaaaattctcagatccag

clpP2_HIII_R CCCAAGCTTtcagtggtggtggtggtggtgggcggtttgcgcggagag

pSYMP-*glnA1* glnA1_Nde_F GGGAATTCCATATGacggaaaagacgcccgac

glnA1_HIII_R CCCAAGCTTtcagtggtggtggtggtggtgaacgtcgtagtacagcgcga

pSYMP-*groEL1* groEL1_Nde_F GGGAATTCCATATGagcaagctgatcgaatacgacgaaac

groEL1_RV_R CCGGATATCtcagtggtggtggtggtggtggtgcgcgtgcccgtggtg

pSYMP-*icl* icl_Nde_F GGGAATTCCATATGtctgtcgtcggcaccccg

icl_HIII_R CCCAAGCTTtcagtggtggtggtggtggtggtggaactggccctcttcgg

pSYMP-Rv0247c Rv0247c_Nde_F GGGAATTCCATATGacgtacagcgcgagtatgcg

Rv0247c_HIII_R CCCAAGCTTtcagtggtggtggtggtggtggcggcgaaacagcttgct

pSYMP-Rv2859c Rv2859c_Nde_F GGGAATTCCATATGgatttgagcgcctctaggtccgat

Rv2859c_HIII_R CCCAAGCTTtcagtggtggtggtggtggtgtcgtggctccgcttggct

pSYMP-*serC* serC_Nde_F GGGAATTCCATATGgccgaccagctcaccccc

serC_HIII_R CCCAAGCTTtcagtggtggtggtggtggtgaagccgctcgaccacccag

pSYMP-*cmaA2* cmaA2_Nde_F GGGAATTCCATATGacgtcacagggcgacacga

cmaA2_HIII_R CCCAAGCTTtcagtggtggtggtggtggtgtttgaccagagtgaactggcagac

***Regulators:***

pSYMP-Rv0042 Rv0042c_Nde_F GGGAATTCCATatgagtgttgtccgctcgattg

Rv0042c_HIII_R CCCAAGCTTTCAGTGGTGGTGGTGGTGGTGcgctcgtttgcgagcaggggagttg

pSYMP-Rv0144 Rv0144_nde_F GGGAATTCCATatgccgcactcttggaccccg

Rv0144_HIII_R CCCAAGCTTTCAGTGGTGGTGGTGGTGGTGggtcggaggccgccagagcg

pSYMP-Rv0158 Rv0158_nde_F GGGAATTCCATatgccatccgacaccagccccaac

Rv0158_HIII_R CCCAAGCTTTCAGTGGTGGTGGTGGTGGTGcgtttccttccgagttccaag

pSYMP-Rv-339c Rv0339c_nde_F GGGAATTCCATatgcagcacagggggtgcaag

Rv0339c_HIII_R CCCAAGCTTTCAGTGGTGGTGGTGGTGGTGacgcctctcatctgcggttaag

pSYMP-Rv0472c Rv0472c_nde_F GGGAATTCCATatggcagagcgtatcccggccg

Rv0472c_HIII_R CCCAAGCTTTCAGTGGTGGTGGTGGTGGTGgacctgcccccaggcaggcacgatc

pSYMP-Rv0576 Rv0576_nde_F GGGAATTCCATatgctggaagtcgcggcagag

Rv0576_HIII_R CCCAAGCTTTCAGTGGTGGTGGTGGTGGTGggtggcggacacgtggcctg

pSYMP-Rv0681 Rv0681_nde_F GGGAATTCCATatggctcgcccggccaaactgag

Rv0681_HIII_R CCCAAGCTTTCAGTGGTGGTGGTGGTGGTGcgttgacgcggtaccaccgtgtgtg

pSYMP-*phoP* phoP_nde_F GGGAATTCCATatgcggaaaggggttgatctc

phoP_HIII_R CCCAAGCTTTCAGTGGTGGTGGTGGTGGTGtcgaggctcccgcagtacgtagccc

pSYMP-*phoY2* phoY2_nde_F GGGAATTCCATatgcggaccgcctaccatg

phoY2_HIII_R CCCAAGCTTTCAGTGGTGGTGGTGGTGGTGgggaaaggcgccggtcgcctgg

pSYMP-Rv0823c Rv0823c_nde_F GGGAATTCCATatgagcaggaggcgggcaatcc

Rv0823c_HIII_R CCCAAGCTTTCAGTGGTGGTGGTGGTGGTGgccacccgaccccatggcgtcc

pSYMP-*embR* embR_nde_F GGGAATTCCATatggctggtagcgcgacagtgg

embR_HIII_R CCCAAGCTTTCAGTGGTGGTGGTGGTGGTGcgtgccgccatgcgtccccg

pSYMP-*whiA* whiA_nde_F GGGAATTCCATatgacgaccgatgtcaaagac

whiA_HIII_R CCCAAGCTTTCAGTGGTGGTGGTGGTGGTGggcgtcttccagcagatcgggc

pSYMP-*moxR1* moxR1_nde_F GGGAATTCCATatgacatcagcaggtgggttc

moxR1_HIII_R CCCAAGCTTTCAGTGGTGGTGGTGGTGGTGccggccgctcgccgcggccgcggcctgcat

pSYMP-Rv1626 Rv1626_nde_F GGGAATTCCATatgaccggccccaccaccgacg

Rv1626_HIII_R CCCAAGCTTTCAGTGGTGGTGGTGGTGGTGggtgtctttgggtgttccgaggg

pSYMP-Rv2358 Rv2358_nde_F GGGAATTCCATatggtgacgtccccctcaacgc

Rv2358_HIII_R CCCAAGCTTTCAGTGGTGGTGGTGGTGGTGtattgcgtcctcaccggcgtgc

pSYMP-*ideR* ideR_nde_F GGGAATTCCATatgaacgagttggttgatacc

ideR_HIII_R CCCAAGCTTTCAGTGGTGGTGGTGGTGGTGgactttctcgaccttgaccgcg

pSYMP-*devS* devS_nde_F GGGAATTCCATatgacaacagggggcctcg

devS-HIII_R CCCAAGCTTTCAGTGGTGGTGGTGGTGGTGctgcgacaacggtgctgacc

pSYMP-Rv3208 Rv3208_nde_F GGGAATTCCATatgagcgatctcgccaagacagc

Rv3208_HIII_R CCCAAGCTTTCAGTGGTGGTGGTGGTGGTGcgagcgggtaagcgggacgtg

pSYMP-*mtrA* mtrA_nde_F GGGAATTCCATatggacaccatgaggcaaag

mtrA_HIII_R CCCAAGCTTTCAGTGGTGGTGGTGGTGGTGcggaggtccggccttgtac

pSYMP-Rv3295 Rv3295_nde_F GGGAATTCCATatggcgacagccagaaggcgg

Rv3295_HIII_R CCCAAGCTTTCAGTGGTGGTGGTGGTGGTGcattcgcgcggttgccatcgc

pSYMP-Rv3583c Rv3583c_nde_F GGGAATTCCATatgattttcaaggtcggagac

Rv3583c_HIII_R CCCAAGCTTTCAGTGGTGGTGGTGGTGGTGagacgcggcggctaaaacctc

pSYMP-Rv3676 Rv3676_nde_F GGGAATTCCATatggacgagatcctggccaggg

Rv3676_HIII_R CCCAAGCTTTCAGTGGTGGTGGTGGTGGTGcctcgctcggcgggccagtc

pSYMP-Rv3833 Rv3833_nde_F GGGAATTCCATatgtcggaaaacagccaccac

Rv3833_HIII_R CCCAAGCTTTCAGTGGTGGTGGTGGTGGTGgcggcgatcgcgagcgcggcggag

pSYMP-*phoH2* NdephoH2f2 ggaattccatatgaccgatacccgcacgtacgtgctc

phoH2-BamHI-r1 aaggatcctcagtggtggtggtggtggtggcgcggcccggtgatctcctcgag
